# Highly Sensitive Closed Loop Enclosed Split Ring Biosensor With High Field Confinement for Aqueous and Blood-Glucose Measurements

**DOI:** 10.1038/s41598-020-60806-9

**Published:** 2020-03-05

**Authors:** Abhishek Kandwal, Tobore Igbe, Jingzhen Li, Yuhang Liu, Sinan Li, Louis W. Y. Liu, Zedong Nie

**Affiliations:** 1 Shenzhen Institutes of Advanced Technology, Chinese Academy of Sciences, Shenzhen, China; 2 Vietnamese-German University, Thu Dau Mot, Binh Duong, Vietnam

**Keywords:** Sensors and probes, Biomedical engineering

## Abstract

This paper presents a highly sensitive closed loop enclosed split ring biosensor operating in microwave frequencies for measuring blood glucose levels in the human body. The proposed microwave glucose biosensor, working on the principle of high field confinement and concentrated energy, has been tested using both *in-vitro* and *in-vivo* methods. This principle allows the sensor to concentrate energy at the surface which results in improved accuracy of measurements. For *in-vitro* measurements, the biosensor has been tested using de-ionized water glucose solutions of different concentrations. The miniaturized micrometer scale biosensor is fabricated over a thin Si-substrate using photolithographic technique. The biosensor has been designed in a way to operate at desired microwave frequencies. Highly confined fields and concentrated energy inside the closed loop line containing the split ring resonators are responsible for the sensitivity enhancement. This new biosensor has obtained a high sensitivity of 82 MHz/mgmL^−1^ within the clinical diabetic range during *in-vivo* testing over the human body. In addition, the subjects (undergoing experiments) steady state has been continuously monitored throughout the experiment which helps in improving the accuracy of the results. The proposed biosensor has further obtained a low detection limit of <0.05 wt.% and can be useful for continuous non-invasive blood glucose monitoring.

## Introduction

Healthy life can be ensured by monitoring human body for heart rate, blood pressure, etc and these need regular checkup for early diagnosis of the diseases. Diabetes is one of the most common chronic diseases that occur due to an imbalance in the glucose levels of the body^1–3^. Diabetes mellitus (DM) is a major cause of mortality and morbidity in every country and low nutrition life made diabetes mellitus one of the most commonly seen diseases among chronic conditions. The diabetic patients suffers from many disorders in their body and the treatment is further more painful. Irrespective of the current available invasive techniques, the non-invasive blood glucose monitoring has attracted lot of attention in the recent years. The need for painless treatment for diabetic patients has become a necessity nowadays^4,5^.

For painless treatment and to measure the blood glucose levels accurately, a new methodology for non-invasive blood glucose measurements is indeed a requirement now. Measuring the blood glucose levels using electromagnetic (EM) waves is one of the most prominent candidates fulfilling the criteria for blood glucose monitoring^6,7^. This technique is based on the assumption that, when the blood glucose level changes, the permittivity of the blood or the muscle tissues change accordingly. The research groups worldwide have considered different approaches for measurements and considered electromagnetic wave sensing as a prominent method. There are other methods also available such as electrochemical sensors or optical spectroscopy but EM wave method has its own advantages in terms of designing, fabrication, ease of use, robustness and cost effectiveness. In^8,9^, the idea of electromagnetic wave sensing mainly in microwave frequencies has been proposed. The research proposed a patch resonator to evaluate the blood glucose levels and study dielectric properties using tissue mimicking phantoms. In^10–12^, the works tried to develop sensors that can efficiently measure the glucose concentrations. They proposed highly sensitive sensors using water-glucose solutions and using blood serum samples. In^13–15^, research groups have been actively involved in finding a suitable methodology and developing glucose sensing system at microwave and millimeter wave frequencies. The research group proposed a sensing system that can measure glucose levels in the human body using transmission measurements at mm-wave frequencies. Simple patch resonators have been used that have not achieved enough sensitivity but the detection limit was good. In^16^, the work has focused mainly on the development of the sensors but the measurements were proof of concept and not actually done on diabetic patients or performed water-glucose solutions instead of blood glucose respectively. Our research group proposed an approach using bio-impedance measurements^17^ and also done work on in-body radio channels for wireless implants^18^. In another work, our research proposed a new methodology to measure blood glucose levels in the human body successfully in millimeter wave frequencies^19^ and the technique is under development for further refinement. The technique used transmission measurements and a new concept was introduced. Most of the research groups are mainly targeting the development of new methodologies and models for investigating the dielectric properties of the blood and using new improved sensors which should be compact, flexible with miniaturized dimensions and high sensitivities.

However, the traditional methods still needs developments and face challenges in terms of low energy concentration, radiation losses, accuracy and reproducibility of the results. To detect the blood glucose levels, the electromagnetic waves should incur minimum losses during their propagation and should have high field concentration so that the beams must be energetic, confined and efficient enough. The high concentration of energy at the sensing area will lead to reduction in the radiation losses that will in turn increase the sensitivity of the surface. The results of our investigation reveals that measurement of blood glucose levels using electromagnetic waves will be accurate if following issues are properly addressed:

1. In microwave frequencies, highly sensitive sensors are required so that the permittivity of blood changes with the glucose concentration within the range between the 75 mg/dl and 200 mg/dl. As discussed above, most of the papers performed solution based glucose measurements and very few papers have done actual on-body glucose measurements.
2. High energy concentration is a factor that can enhance the sensitivity of the sensors. The sensors can be designed and optimized in a way that they achieve this property.
3. Detection limit be should be low. Along with the detection of high glucose levels, the sensors should be able to detect low glucose levels also.

To overcome the above-mentioned issues, the proposed blood glucose measurements were conducted at microwave frequencies with the help of a closed loop enclosed split ring based sensor. This biosensor has effectively concentrated energy on the surface by generating highly confined fields. The split rings, also known as metamaterials, will help in concentrating the energy and are coupled to the closed loop structure thereby enhancing the field confinement. In recent years, metamaterial based sensors have become very popular and has huge potential. Metamaterials used in sensor applications have been designed for high frequency operation by a significant reduction in the overall size. Microwave sensors based on metamaterials are useful in the field of chemical analytic and medical diagnostics due to a wide range of beneficial properties especially for the development and realization of compact and highly sensitive devices. The split-ring resonator based designs operating in the microwave region combines the features of a microwave resonator and metamaterial for the development of compact and cost efficient sensing devices^20–25^. In^20^, authors shown the advantage of using split rings as biosensors but not for glucose measurements. In^22,23^, the proposed resonators measures the aqueous glucose concentrations using a simple epsilon negative resonator and double split ring resonators (circular shaped) but no invivo experiments were conducted. In^24,25^, single split ring resonator and a modified metamterial resonator was used to determine aqueous glucose concentrations but the sensitivity obtained was low. These research groups have performed *in-vitro* glucose measurements but direct *in-vivo* testing was not done. This maybe due to low achieved sensitivity in the glucose solutions.

The proposed closed loop split ring glucose sensor has combined the features of split ring resonators and the concept of highly concentrated energies to successfully achieve high sensitivity and low detection limit in both *in-vitro* and *in-vivo* measurements. A new closed loop enclosed split ring sensor has been designed. The enclosed double split rings coupled with each other and also to the closed loop microstrip line results in enhanced concentration of energies around the surface. The measurements have been performed over human subjects with steady physical state to improve the accuracy. The deionized water-glucose measurements and tests over human body provide good results and the glucose levels have been adequately detected.

## Fabrication of the Biosensor, Methods and Principle

**Figure 1:**
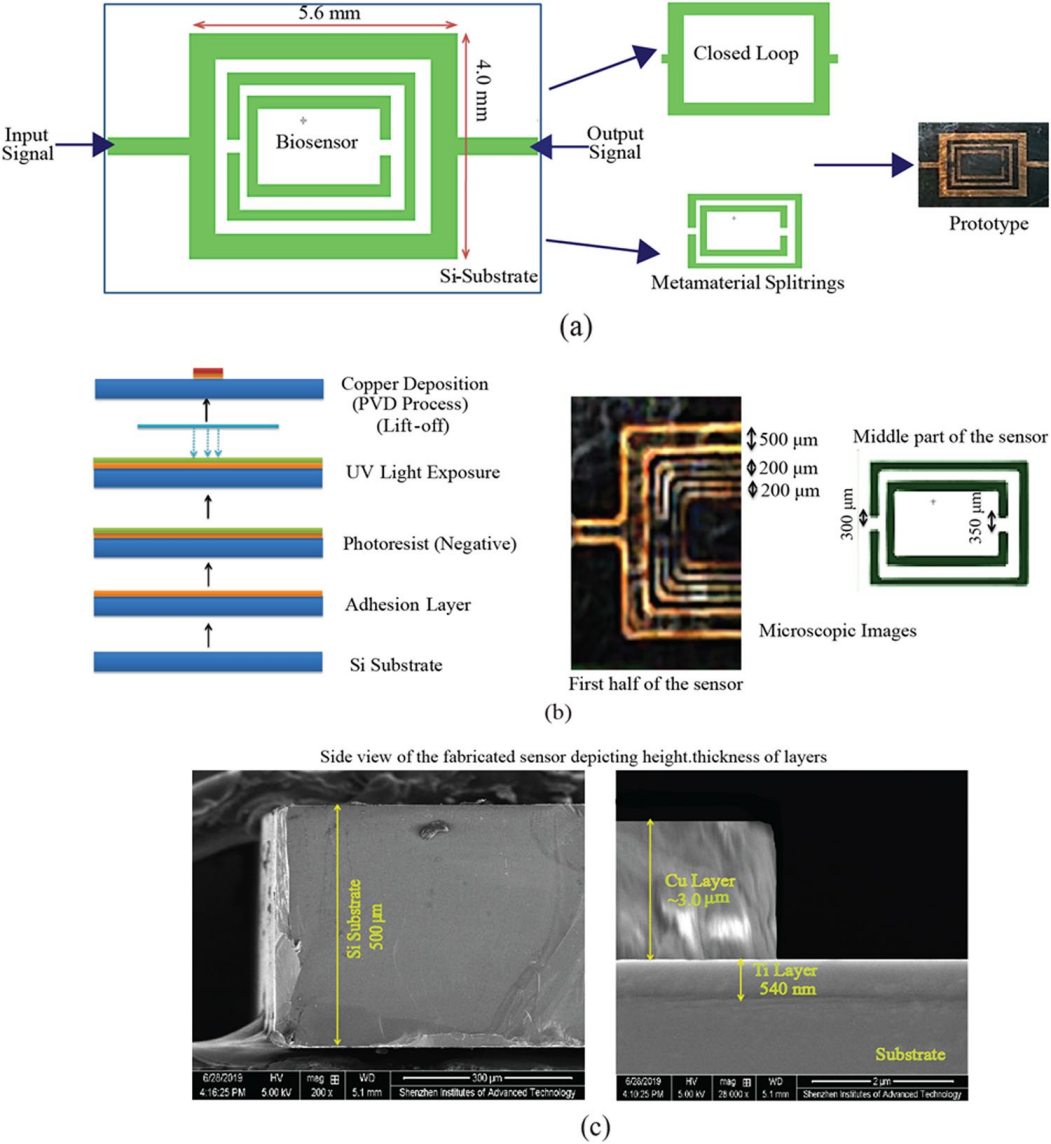
Biosensor: (**a**) Schematic of Proposed Sensor Design (**b**) Fabrication Process and microscopic images of sensor (**c**) Scanning Electron Microscope (SEM) Images (showing thickness of layers).

**Figure 2:**
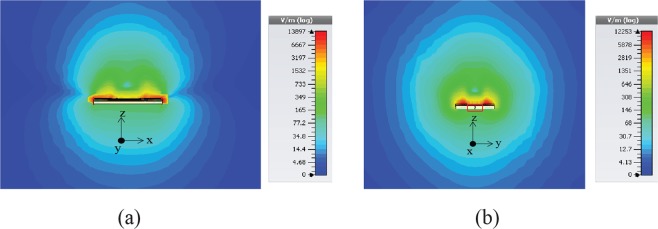
Field distribution: High Field Confinement.

The proposed glucose sensor consists of closed loop enclosed split ring resonators and the geometry is shown in Fig. 1(a). The sensor is designed on a thin silicon substrate of height 500 micrometers. The length of main body of the sensor is 5.6 mm and width 4.0 mm. The sensor is connected to microstrip lines of 50 ohm on both sides viz. input and output terminals. The overall dimension of the sensor design is (9.6 mm × 6.0 mm). The width of the closed loop microstrip line is 500 micrometers. The spilt ring resonators have line width of 200 micrometers each. The resonator split gap is 300 micrometers and 350 micrometers in each case respectively. The split ring resonators are coupled to each other and with the closed loop line enclosing the rings. The fabrication process is shown in detail in Fig. 1(b). The proposed biosensor has been fabricated using photolithography technique and physical vapor deposition (PVD) technique to deposit copper over a silicon (Si) substrate. The dielectric constant of the substrate used is 11.9. The silicon substrate is first prepared and covered with an adhesion layer over it. A negative photoresist is then spin coated over the substrate and after applying mask has been exposed to UV light. The area exposed UV light becomes insoluble in photoresist developer and we can remove the unexposed resist area from the substrate when developed. Copper (Cu) metal layer of thickness 3 microns is finally deposited using physical vapour deposition technique and finally get Cu sensor structure by removing the resist by lift of process. Figure 1(c) shows the scanning electron microscope (SEM) images of the sensor design. The thickness of each layer can be seen clearly in the images. Figure 2 depicts the field distribution of the proposed sensing region. As a sensitivity enhancement principle, the fields should be highly confined on and around the sensing region of the sensor which is one of the requirements of our proposed methodology. As can be clearly seen from the figure, the energy is mostly concentrated on the desired area. Figure 2(a,b) shows the energy concentration and field confinement at different correctional axis. The coupled meta-rings enclosed in a closed loop line accommodates most of the energy thereby making the area highly sensitive for sensing of the glucose concentrations effectively.

**Figure 3:**
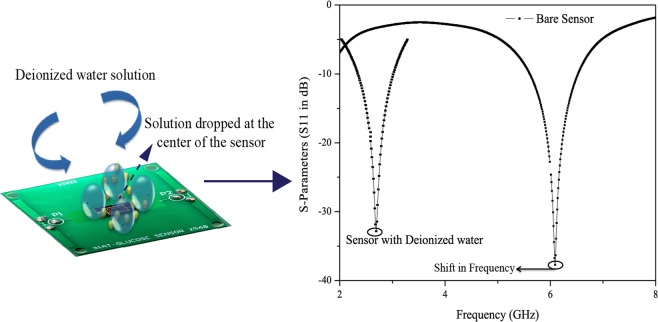
Printed Circuit Board (PCB) Packaging for Invitro Analysis and Resonance Shift Concept.

 Figure 3 shows the PCB (printed circuit board) packaging of the proposed glucose sensor for *in-vitro* measurements. The figure also shows the preliminary results of the sensor for two cases: bare resonator and with de-ionized water. As can be seen from the figure, resonant frequency has been shifted from 6.1 GHz to 2.8 GHz when de-ionized water dropped using a finnpippette over the sensing region. A noticeable change of 5 dB in the return loss can also be seen.

## Experimental Measurements in Controlled Environment

**Figure 4:**
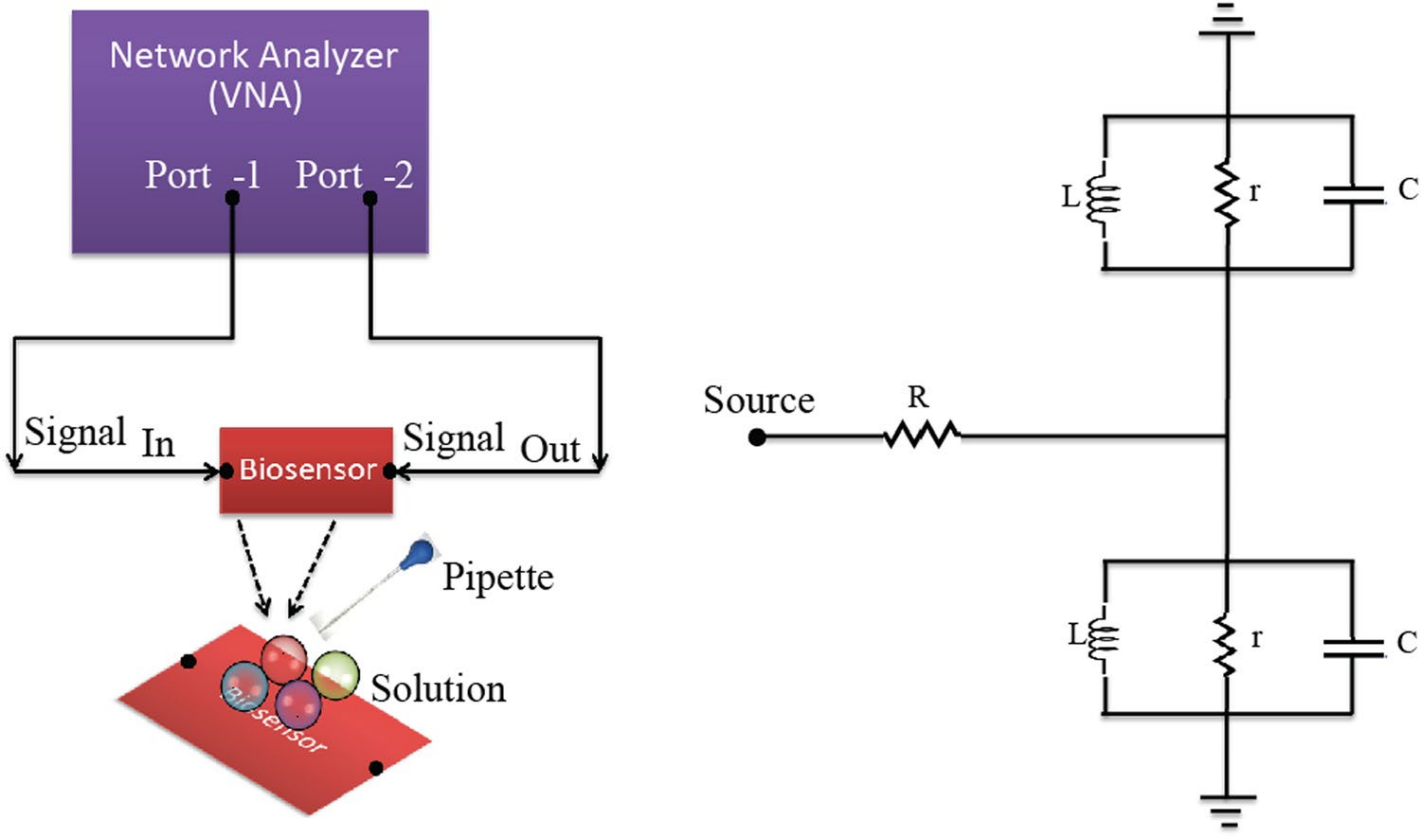
Schematic of measurement setup for Invitro tests and equivalent circuit diagram of the sensor.

In this work, the blood glucose levels were monitored using electromagnetic waves in a setup as illustrated in Fig. 4. In Fig. 4, the network analyzer was used to measure the scattering parameters (S11) over the 500 kHz–12 GHz frequency range. It also shows the equivalent circuit diagram of the proposed sensor over the substrate where L (38.5 pH) is inductance, C is capacitance, r (100 ohm) and R (13 ohm) are resistances. All the measurements were performed in a closed clean room with normal room temperature and with minimum noise levels. The *in-vivo* experimental setup also takes into account the physical condition of a person. As per the studies, the physical movements of a human body are also important to analyse blood glucose levels. Therefore in order to achieve more accuracy during the measurements, steady state of a person has been tested before and during performing the experiment over the subjects in case of *in-vivo* tests. The experimental diagram has been shown in the Supplementary file as Fig. S1.

### *in-vitro*: Sensing in deionized water-glucose solutions using resonance frequency shift method

**Figure 5:**
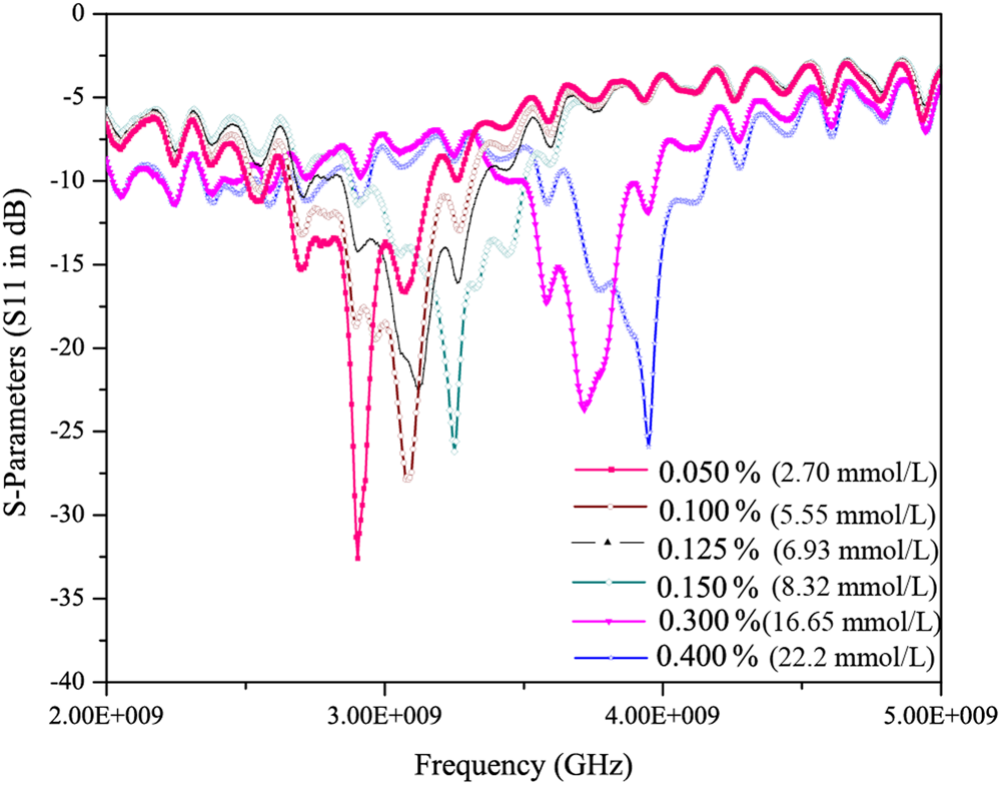
Invitro S11 measurements: Shift in the resonant frequencies.

An aqueous glucose standard solution was prepared using a mixture of deionized-water and D-glucose powder. Glucose concentrations of 0.050, 0.100, 0.125, 0.150, 0.300, and 0.400 percent by weight (2.7 mmol/L, 5.55 mmol/L, 6.93 mmol/L, 8.32 mmol/L, 16.65 mmol/L, 22.2 mmol/L) were prepared by dissolving glucose with a solution of deionized water. This range of 0.050–0.400 percent is suitable for clinical test of glucose because the normal range of blood-glucose level for a diabetic patient ranges from 0.075 to 0.200 percent (i.e. 4.1 mmol/L to 11.1 mmol/L). To determine the analytical performance of the biosensor, the S-parameters of the bare resonator was first characterized over a frequency range from 0.1 to 12 GHz using the Agilent vector network analyzer (VNA). During the measurement, a portion of the electromagnetic wave from port 1 of the network analyzer was incident onto the device under test (DUT) and reflected back to port 1. The measured S11 parameters can be used to determine how much electromagnetic energy was reflected. The remaining portion of the electromagnetic wave from the network analyzer was either transmitted to the load or dissipated by the device under test (DUT) as heat. Figure 5 shows *in-vitro* measurements for different glucose concentrations as mentioned above. The resonant frequencies have been observed to be shifted with the variation of deionized water-glucose concentrations. In a range of concentrations from 0.050 percent to 0.400 percent by weight (2.7 mmol/L to 22.2 mmol/L), the resonant frequencies are 2.85 GHz, 3.05 GHz, 3.1 GHz, 3.2 GHz, 3.7 GHz and 3.95 GHz respectively. This noticeable shift in the resonant frequencies is due to the change in permittivity with different glucose concentrations. The shift in frequency is directly proportional to the glucose concentrations and inversely proportional to the permittivity. The sensitivity of the proposed glucose biosensor is found to be 350 MHz/mgmL^−1^ within the clinical diabetic range. The proposed glucose sensor has also low detection limit and has been able to successfully measure very low glucose concentrations less than 0.050 percent by weight. The measurements have been repeated atleast ten times and each time almost similar variations and shifts have been observed. Some variations existed may be due to slight handling issues during measurements. The results for five chosen measurements have been shown in the Supplementary Section as Fig. S2.

**Figure 6:**
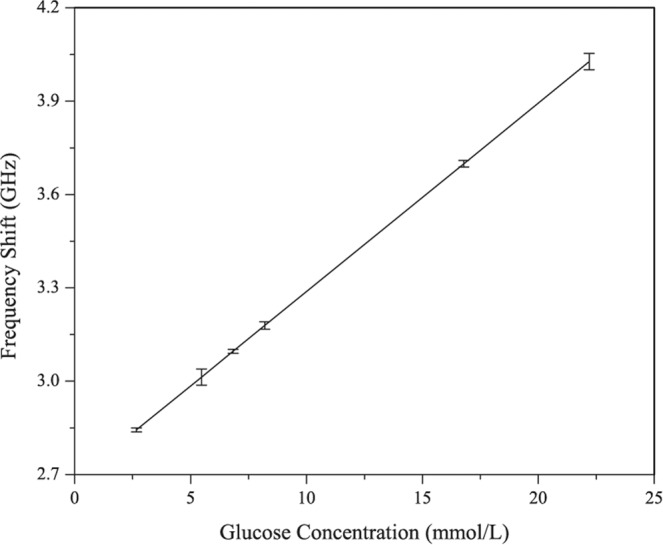
Linear fit (Invitro).

Within this tested range, the return loss is changed significantly from 33.5 dB to 26 dB. The measurements have been repeated number of times and a good reproducibility has been achieved. Figure 6 shows the linear fitting of the results obtained in Fig. 5 for *in-vitro* measurements with error bar. A linear correlation between the glucose values and observed shift in resonant frequencies has been observed. Some points don’t lie exactly in the linearity range but a near linear relationship has been obtained. The graph has also considered the error bar during linear fitting as shown in the figure.

### *in-vivo*: Human body clinical investigations in controlled environment

The proposed glucose sensor has further been tested for real time *in-vivo* blood glucose monitoring at the Center of Biomedical and Health Engineering, Shenzhen Institutes of Advanced Technology, Chinese Academy of Sciences. Five healthy male volunteers have taken part in the *in-vivo* tests and a confirmed consent has been obtained from them before performing the experiments. The experiments were approved by Shenzhen Institutes of Advanced Technology, Chinese Academy of Sciences ethics committee and all experiments were performed in accordance with the relevant guidelines and regulations. For each volunteer, the measurements have been repeated 3–5 times in order to achieve accuracy in the results.

**Figure 7:**
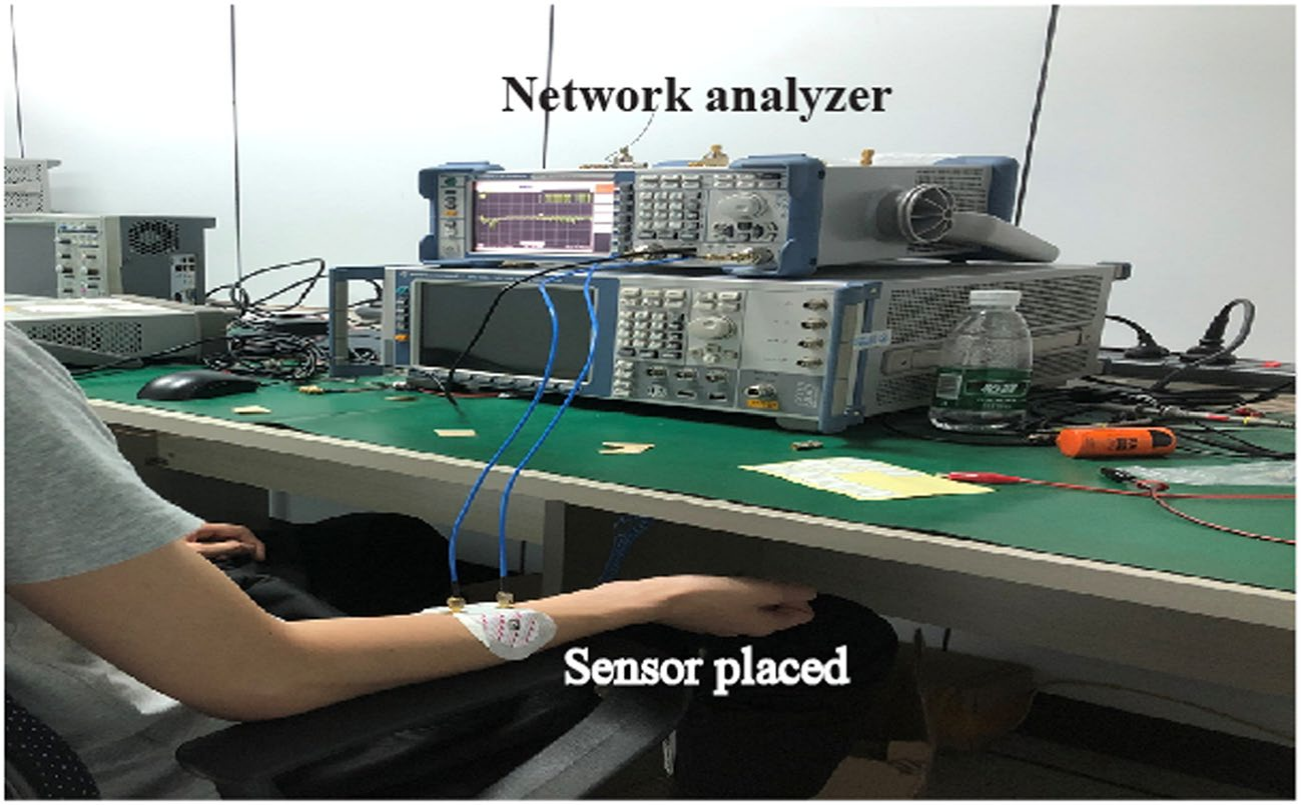
Experimental setup for blood glucose measurements.

**Figure 8:**
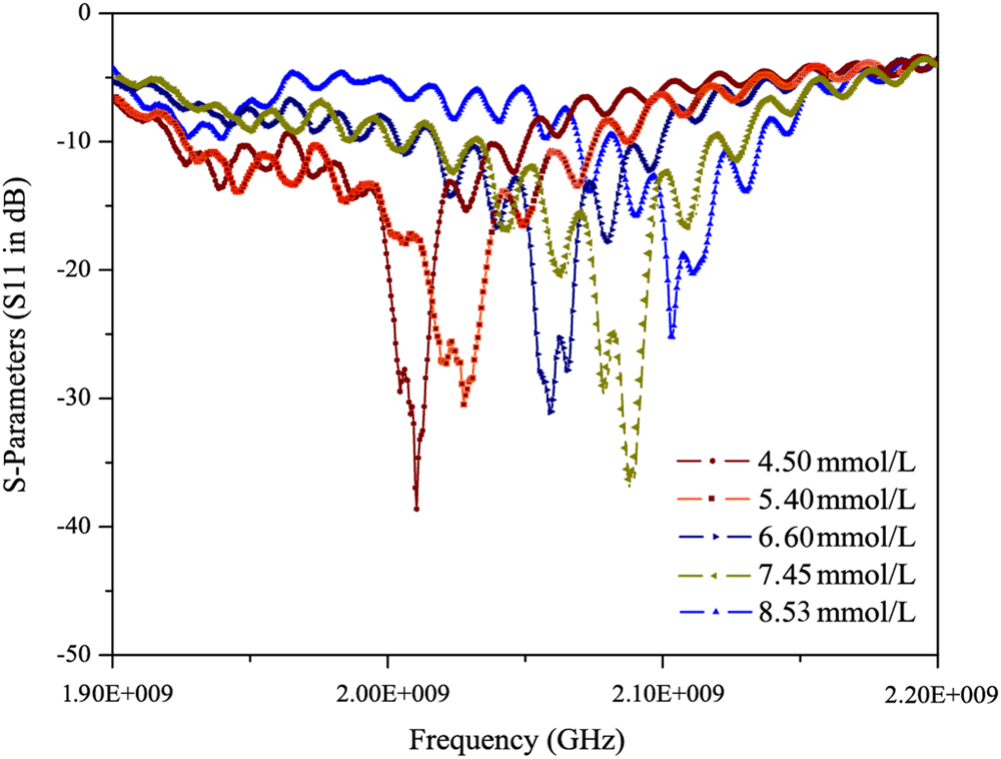
Invivo S11 measurements: Shift in the resonant frequencies.

**Figure 9:**
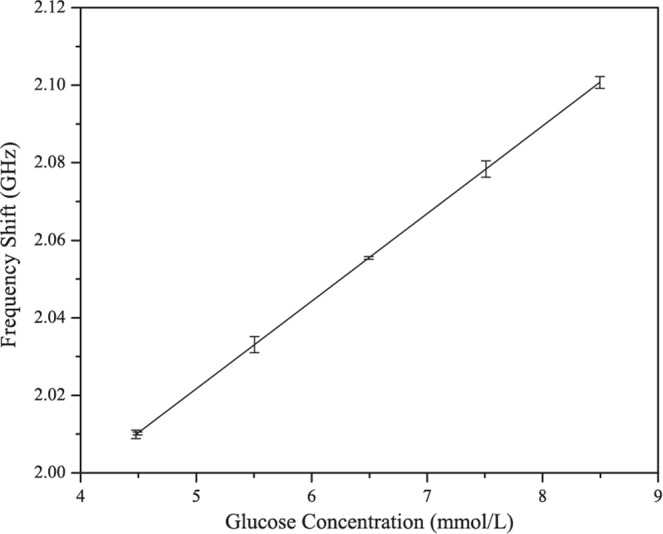
Linear fit (Invivo).

 Figure 7(a) shows the *in-vivo* experimental measurement setup in a clean room. The proposed sensor is attached on the right arm of the volunteer (subject) so that there should be no movement during measurements. Further the subject is also continuously monitored for steady state physically. This will further improve the accuracy and reduce the errors incurred due to physical movements in the body. The sensor attached to the right arm of the subject is connected to the vector network analyzer. Standard SMA connector were used to connect sensor and the network analyzer. The experiment to investigate the influence of glucose levels on the frequency shift (S11 parameter) is performed with oral glucose tolerance test (OGTT). The experiment started at 9:28 AM and throughout the experiment the patient sat in a comfortable position with no movement or as minimum as possible. Now exactly 30 minutes into the experiment, the volunteer was given 75 gm of anhydrous glucose solution and at regular intervals the index finger on the left hand is pricked to obtain a pinch of blood for the test with a blood strip device called ACCU-CHEK meter. Simultaneously, at 25-minutes interval S11 parameters were measured with the glucose sensor connected to a vector network analyzer. Figure 8 shows *in-vivo* measurements in terms of frequency shifts for different glucose concentrations. Within the clinical diabetic range, a marked shift in the resonant frequencies has been observed. At glucose concentrations of 4.5 mmol/L, 5.4 mmol/L, 6.6 mmol/L, 7.45 mmol/L, 8.53 mmol/L; the resonant frequencies are 2.02, 2.035, 2.055, 2.08, 2.102 respectively. The proposed glucose sensor has achieved good performance during clinical investigations and observed a high sensitivity of 82 MHz/mgmL^−1^. The shift in the frequencies follow a similar trend as in case of *in-vitro* measurements and varies directly proportional to the glucose concentrations. Figure 9 shows the linear fitting of the results obtained with error bar in Fig. 8 for *in-vivo* measurements. A linear correlation between the glucose values and observed shift in resonant frequencies has been observed. For comparison purpose, results for all five volunteers have been shown in Fig. S3 of Supplementary file. Slight variations in the linear dependency have been observed for different person, but the overall frequency shifts have been observed to be almost similar. For similar variations (i.e. from 4.5 mmol/L to 8.53 mmol/L) in both Figs. 6 and 9, the invitro experiment shows a frequency shift of 250 MHz/mgmL^−1^ and the invivo shows a frequency shift of 82 MHz/mgmL^−1^. Additionally, the main point is to achieve frequency shift in both cases along with near linear correlation for varying glucose concentrations.

Further another point for discussion is the heating effect. It is to be understood that the microwave power of the signal from ports is no more than 0 dbm. Such a low power has virtually zero localized heating effect. Even if the sensor is patched onto the skin for long time, the incoming microwave is simply not enough to generate heating.

For confirmation of specificity, we first performed invitro tests by adding different analytes with glucose in the solution that can affect the measurements such as Na, Cl, calcium, vitamins, and fructose. As a result, it is found that the sensor has again provided similar frequency shifts in the proposed frequency band/window. This shows that the sensor is sensitive only to the glucose. Therefore during invivo tests also, we can analyze that the shifts observed are due to change in glucose concentrations. Further for invivo tests, the subject may also have other analytes in his/her body. As a result the linear dependency varies a little for different person (as shown in Figs. S2 and S3 added in the Supplementary Section).

 Table 1 indicates the comparison between previous published glucose sensors and the proposed sensor. The proposed glucose biosensor has used reflection based detection technique for *in-vitro* and *in-vivo* tests. Most of the microwave based glucose have not performed direct on-body *in-vivo* tests as also compared. The solution based sensing has achieved less sensitivity than the proposed one. In^11^ the sensitivity is very high but the invivo tests were not done. In^13^ invivo experiments were performed but transmission based techniqe was adopted and simple patch antenna was used which may have less efficiency. The proposed biosensor has also an advantage of reduced number of metallic layers as compared to other works. It reduce the fabrication cost pertaining to less complex design. The biosensor has also a significantly lower detection limit, a wide linear detection range and has been tested for direct on-body investigations. The low detection limit refers to the case where the sensor detects glucose levels below 4 mmol/L (or below 75 mg/dL or <0.075 wt.percent). In our case the sensor successfully detects very low glucose concentrations during invitro tests i.e. 0.05 wt.percent. We have included this parameter in the Table 1 with lowest value observed firmly i.e. 0.05 wt.percent or 50 mg/dL or 4 mmol/L.

**Table 1:** Comparison with the counterparts.

Reference	Method	Technique	On-body	Sensitivity (Solution)	Sensitivity (Invivo)	Detection
^10^	Solution,serum	Reflection	No	199 MHz/mgmL^−1^	—	0.03 *μ*M
^11^	Solution,serum	Reflection	No	978.7 MHz/mgmL^−1^	—	0.019 *μ*M
^12^	Solution,serum	Reflection	No	108 MHz/mgmL^−1^	—	0.606 *μ*M
^13^	*in-vivo*	Transmission	Yes	—	Δ = 0.5–3 dB	0.025 wt%
^16^	Solution	Transmission	No	—	—	—
^20^	Solution	Transmission	No	Δ = 120 MHz	—	—
^22^	Solution	Reflection	No	200 MHz(/100mg/mL)	—	—
^24^	Solution	Transmission	No	0.174 MHz/mg/mL	—	—
^25^	Solution	Transmission	No	37 MHz (/30mg/dL)	—	—
Our work	*in-vivo*	Reflection	Yes	350 MHz/mgmL^−1^	82 MHz/mgmL^−1^	< 0.05 wt%

The findings of this work have proven the fact that, at microwave frequencies, there exists a near-linear relationship between the S11 parameters and the blood glucose concentrations. These findings consistently agree with the conclusions drawn by some other research groups who have successfully measured blood glucose concentrations at around the frequency range from 1 GHz to 12 GHz or in other microwave/mm-wave frequencies.

## Conclusions

The proposed glucose sensor has effectively measured blood glucose concentrations in deionized water-glucose solutions and during human on-body investigations. A high sensitivity has been obtained along with a low detection limit resulting from the higher concentration of the energy around the sensing region. This sensitivity enhancement method is very effective and suitable for non-invasive blood glucose measurements. The steady state criteria has also been taken care of during the experiments and the subjects have been continuously observed for physical movements that can affect the accuracy of the measurements directly or indirectly.

### Experiments involving human participants

Author confirms that informed consent was obtained from all the volunteers and Shenzhen Institutes of Advanced Technology, Chinese Academy of Sciences ethics committee approved the human clinical investigation.

## Supplementary information

Supplementary Information.

## References

[1] Organization, W. H. *World Health Day Beat diabetes* (2016).

[2] Standards of medical care in diabetes abridged for primary care providers. *Clinical Diabetes* **37**, 11–34 (2019). 10.2337/cd18-0105 PMC6336119 30705493

[3] Jia, W. Continuous glucose monitoring. *Springer Nature Singapore Pte Ltd. and Shanghai Scientific and Technical Publishers* (2018).

[4] Elsheakh, D. Non-invasive electromagnetic biological microwave testing: Microwave systems and applications. *IntechOpen* (2017).

[5] Lipani L (2018). Non-invasive, transdermal, path-selective and specific glucose monitoring via a graphene-based platform. Nature Nanotechnology.

[6] Ishimaru, A. Electromagnetic wave propagation, radiation, and scattering: From fundamentals to applications. *John Wiley and Sons* (2017).

[7] Karacolak, T., Moreland, E. C. & Topsakal, E. Cole cole model for glucose dependent dielectric properties of blood plasma for continuous glucose monitoring. *Microwave and Optical Technology Letters* **55** (2013).

[8] Yilmaz T, Foster R, Hao Y (2014). Broadband tissue mimicking phantoms and a patch resonator for evaluating noninvasive monitoring of blood glucose levels. IEEE Transactions on Antennas and Propagation.

[9] Yilmaz T, Foster R, Hao Y (2014). Towards accurate dielectric property retrieval of biological tissues for blood glucose monitoring. IEEE Transactions on Microwave Theory and Techniques.

[10] Nam-Young, K.*et al*. Rapid, sensitive, and reusable detection of glucose by a robust radiofrequency integrated passive device biosensor chip. *Scientific Reports* **5**, 10.1038/srep07807 (2015). 10.1038/srep07807 PMC4295091 25588958

[11] Adhikari, K. K. & Kim, N. Y. Ultrahigh-sensitivity mediator-free biosensor based on a microfabricated microwave resonator for the detection of micromolar glucose concentrations. *IEEE Transactions on Microwave Theory and Techniques* **64** (2016).

[12] Kim NY, Dhakal R, Adhikari KK, Kim ES, Wang C (2015). A reusable robust radio frequency biosensor using microwave resonator by integrated passive device technology for quantitative detection of glucose level. Biosensors and Bioelectronics.

[13] Saha, S. *et al*. A glucose sensing system based on transmission measurements at millimetre waves using microstrip patch antennas. *Scientific Reports* **7** (2017). 10.1038/s41598-017-06926-1 PMC5537249 28761121

[14] Garcia, H. C. *et al*. Reflection and transmission measurements using 60 ghz patch antennas in the presence of animal tissue for non-invasive glucose sensing. *10th European Conference on Antennas and Propagation* 1–3 (2016).

[15] Saha, S. *et al*. Evaluation of the sensitivity of transmission measurements at millimeter waves using patch antennas for non-invasive glucose sensing. *10th European Conference on Antennas and Propagation* 1–4 (2016).

[16] Juan C (2019). Concentration measurement of microliter volume water glucose solutions using q factor of microwave sensors. IEEE Transactions on Instrumentation and Measurement.

[17] Li J (2018). An approach for noninvasive blood glucose monitoring based on bioimpedance difference considering blood volume pulsation. IEEE Access.

[18] Li J, Nie Z, Liu Y, Wang L, Hao Y (2017). Characterization of in-body radio channels for wireless implants. IEEE Sensors Journal.

[19] Liu, L. W. *et al*. Non-invasive blood glucose monitoring using a curved goubau line. *Electronics* **8** (2019).

[20] Lee H-J, Yook J-G (2008). Biosensing using split-ring resonator at microwave regime. Applied Physics Letters.

[21] Kim J, Babajanyan A, Hovsepyan A, Lee K, Friedman B (2008). Microwave dielectric resonator biosensor for aqueous glucose solution. Review of Scientific Instruments.

[22] RatneshKumari PNP, Yadav R (2018). An eng resonator-based microwave sensor for the characterization of aqueous glucose. J. Phys. D: Appl. Phys.

[23] Choi H (2015). Design and in vitro interference test of microwave noninvasive blood glucose monitoring sensor. IEEE Transactions on Microwave Theory and Techniques.

[24] Camli B (2016). A microwave ring resonator based glucose sensor. Procedia Engineering.

[25] Islam M, Hoque A, Almutairi A, Amin N (2019). Left-handed metamaterial-inspired unit cell for s-band glucose sensing application. Sensors.

