# Supplementary material for: Highly Sensitive Closed Loop Enclosed Split Ring Biosensor With High Field Confinement for Aqueous and Blood-Glucose Measurements
Source: Sci Rep. 2020 Mar 5;10:4081. doi: 10.1038/s41598-020-60806-9 (PMC7058086; doi:10.1038/s41598-020-60806-9)
Supplement: Supplementary file 1 — Supplementary Information. [file 41598_2020_60806_MOESM1_ESM.pdf]

Highly Sensitive Closed Loop Enclosed Split Ring Biosensor With High Field Confinement for Aqueous and Blood-Glucose Measurements

Abhishek Kandwal[1], Tobore Igbe[1], Li Jingzhen[1], Yuhang Liu[1], Li Sinan[1], Louis WY Liu[2], and Nie Zedong[1*]

[1]A. Kandwal, T. Igbe, L. Jingzhen, Y. Liu, L. Sinan and N. Zedong are with Shenzhen Institutes of Advanced Technology, Chinese Academy of Sciences, Shenzhen, China
[2]L WY Liu is with Vietnamese-German University, Vietnam
Correspondence should be addressed to: zd.nie@siat.ac.cn

Supplementary Results

For more calibrations before and during the experiments that include physical and physiological states, we have also done more experiments. For this purpose, we have used BIOPAC system for analysis. The picture of the experiment is shown below in fig. S1. It has been observed that person under test were in complete resting state physiologically also. This process will further help in achieving more accuracy in the results.

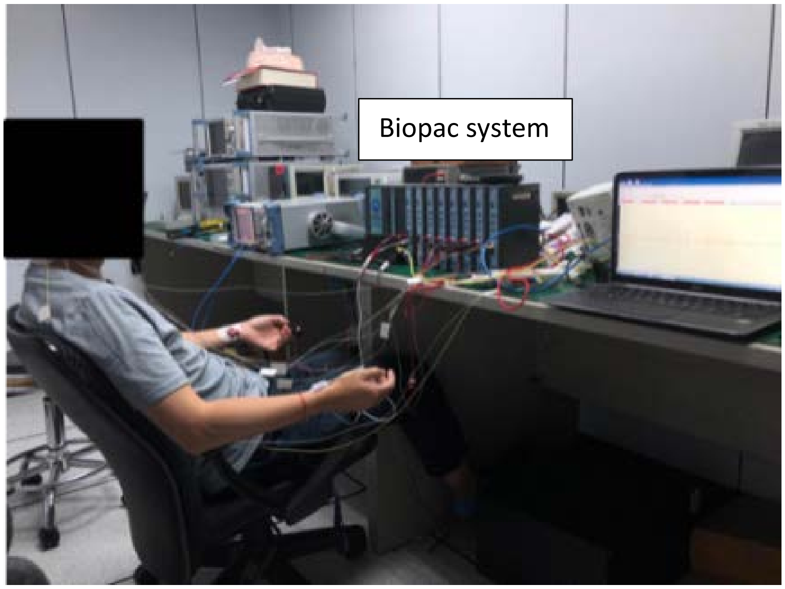

Fig. S1: Physiological steady state

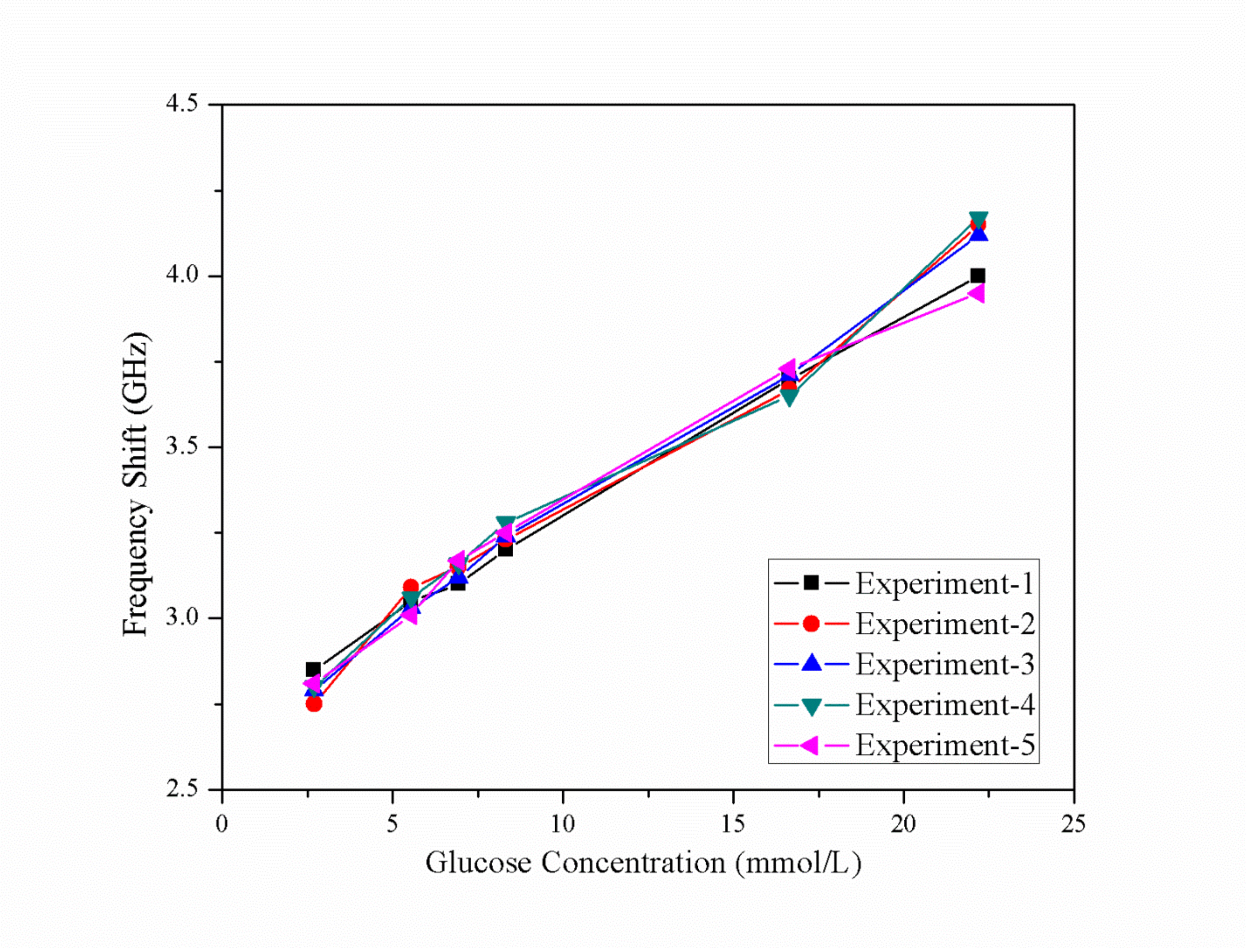

Fig. S2: Comparison showing multiple experiments done during in-vitro measurements.

Fig. S2 shows the comparison of multiple experiments done during the in-intro measurements. Five measurement results have been plotted in the graph above. It has been observed that almost similar kinds of shifts in the frequency have been observed during different measurements. The resonance frequencies varied a little but the change is very minute (maybe due to handling issues during experiments) with same frequency shifts.

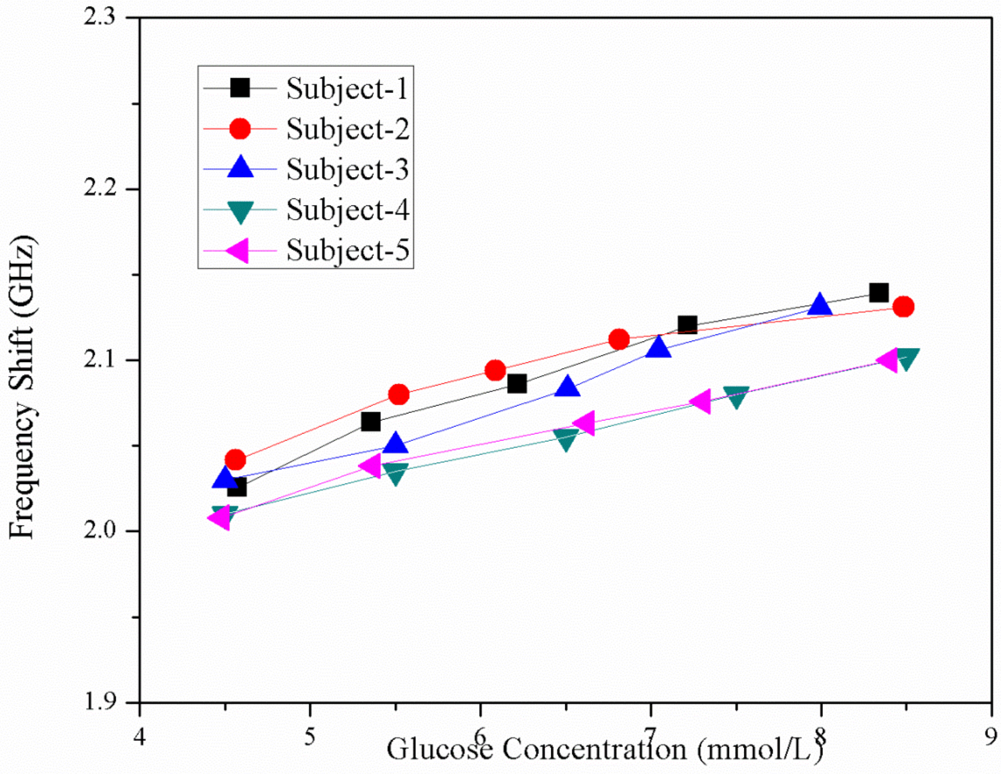

Fig. S3: Comparison showing experiments done on five volunteers during in-vivo measurements.

Fig. S3 shows the comparison of in-vivo experiments done on five volunteers. As we can see that some frequency shifts have been observed which are legitimate because each person conditions can vary. However the shift in the resonant frequencies which is the main criteria has still been observed with high accuracy. Within clinical diabetic range of blood glucose in the human body (shown in fig. S3 also), the total shift in the frequencies is almost same i.e. around 80-85 MHz.
